# Supplementary material for: Wastewater surveillance for Salmonella Typhi and its association with seroincidence of enteric fever in Vellore, India
Source: PLoS Negl Trop Dis. 2025 Mar 3;19(3):e0012373. doi: 10.1371/journal.pntd.0012373 (PMC11896026; doi:10.1371/journal.pntd.0012373)
Supplement: S1 Table — (DOCX) [file pntd.0012373.s001.docx]

**S1 Table.** Physico-chemical characteristics of wastewater – between-site and within-site variation.

| ***Variable*** | ***Median (IQR)*** | ***Between site variation*** | | ***Within-site variation (repeated)*** | |
| --- | --- | --- | --- | --- | --- |
|  |  | ***F Statistic*** | ***p Value*** | ***F Statistic*** | ***p Value*** |
| Temperature (ºC) | 27.8 (26.3 - 28.9) | 0.97 | 0.518 | 20.66 | < 0.001 |
| pH | 7.2 (7.08 - 7.43) | 1.63 | 0.011 | 6.22 | 0.013 |
| Oxidative reductive potential (mV) | -234.5 (-299.5 to -150.6) | 5.17 | <0.001 | 23.71 | < 0.001 |
| Dissolved oxygen (%saturation) | 39.8 (28.9 - 51.1) | 3.07 | <0.001 | 27.63 | < 0.001 |
| Total dissolved solids (mg/L) | 582.5 (452 -754) | 1.87 | <0.001 | 66.59 | < 0.001 |
| Turbidity (NTU) | 107.5 (67.7 - 160) | 5.68 | <0.001 | 8.70 | 0.003 |

IQR – interquartile range; mV – millivolt; mg/L – milligrams/litre, NTU – nephalometric turbidity unit
