# Supplementary material for: Wastewater surveillance for Salmonella Typhi and its association with seroincidence of enteric fever in Vellore, India
Source: PLoS Negl Trop Dis. 2025 Mar 3;19(3):e0012373. doi: 10.1371/journal.pntd.0012373 (PMC11896026; doi:10.1371/journal.pntd.0012373)
Supplement: S2 Table — (DOCX) [file pntd.0012373.s002.docx]

**S2 Table.** Serosurvey participants and seroincidence by catchment areas of sampling sites.

| ***Location ID*** | ***Catchment stratification*** | ***Number of serosurvey participants*** | ***Seroincidence/ 100py*** | ***95% CI*** |
| --- | --- | --- | --- | --- |
| 106 | 1 | 45 | 18.7 | 12.08 – 28.90 |
| 108 | 1 | 125 | 14.3 | 11.19 – 18.40 |
| 110 | 1 | 34 | 24.6 | 15.47 – 39.22 |
| 111 | 1 | 23 | 16.1 | 9.30 – 27.74 |
| 112 | 1 | 31 | 12 | 6.81 – 21.03 |
| 120 | 1 | 20 | 8.6 | 4.15 – 17.79 |
| 122 | 1 | 216 | 10.3 | 8.36 – 12.77 |
| 123 | 1 | 82 | 4.2 | 2.64 – 6.72 |
| 127 | 1 | 27 | 8.2 | 4.14 – 16.37 |
| 130 | 1 | 71 | 6.1 | 3.88 – 9.69 |
| 137 | 1 | 53 | 10.4 | 6.93 – 15.51 |
| 139 | 1 | 210 | 11.6 | 9.44 – 14.18 |
| 143 | 1 | 61 | 7.9 | 5.28 – 11.74 |
| 114 | 2 | 103 | 9.5 | 6.98 – 12.82 |
| 116 | 2 | 494 | 8.3 | 7.13 – 9.56 |
| 145 | 2 | 184 | 20.6 | 16.82 – 25.31 |
| 149 | 2 | 101 | 9.7 | 7.15 – 13.13 |
| 140 | 3 | 887 | 10.3 | 9.25 – 11.36 |
| 141 | 3 | 725 | 10.2 | 9.13 – 11.46 |
| 142 | 3 | 1172 | 10.5 | 9.56 – 11.43 |
