# Supplementary figures and images for: Wastewater surveillance for Salmonella Typhi and its association with seroincidence of enteric fever in Vellore, India
Source: PLoS Negl Trop Dis. 2025 Mar 3;19(3):e0012373. doi: 10.1371/journal.pntd.0012373 (PMC11896026; doi:10.1371/journal.pntd.0012373)

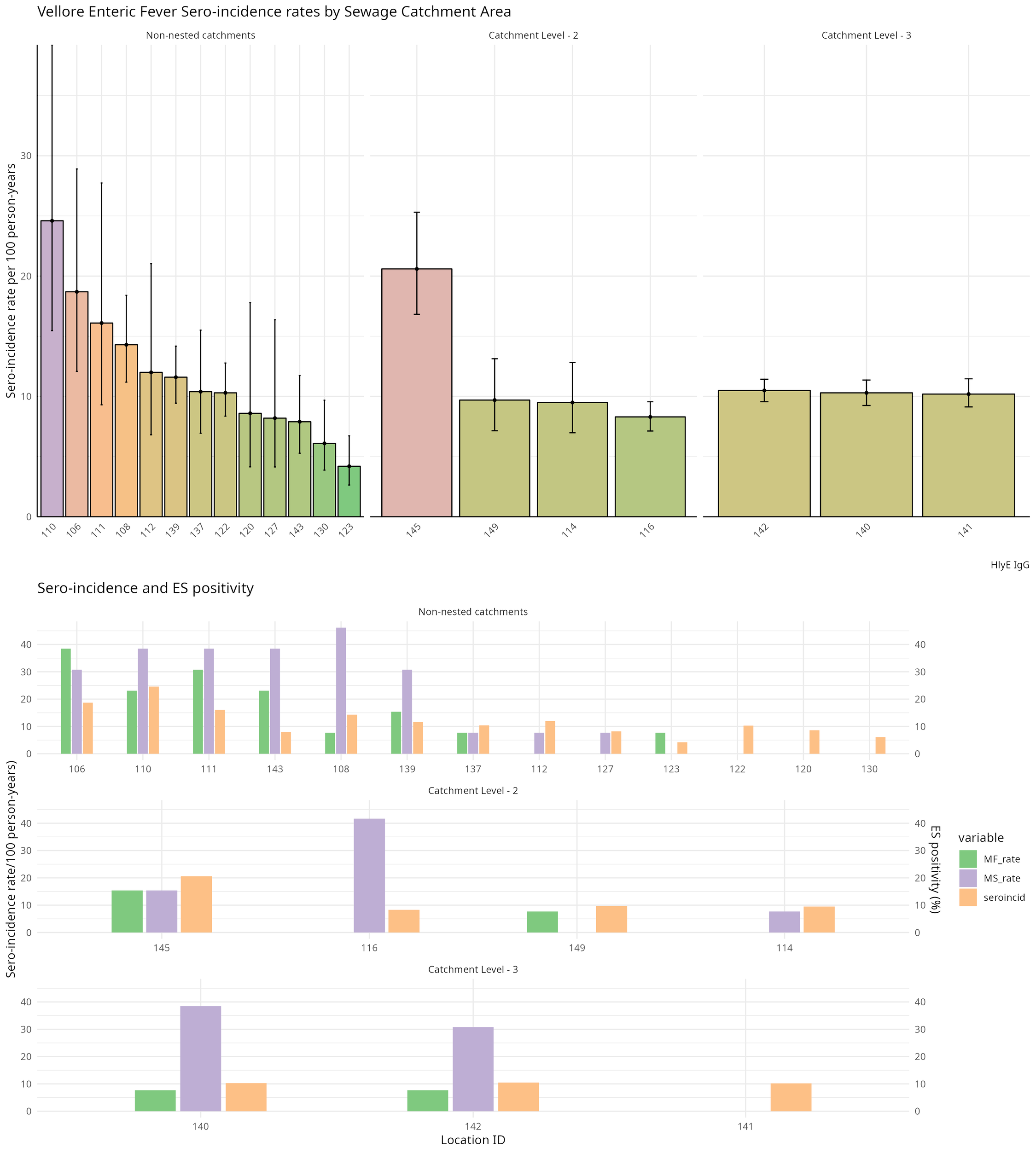

Supplement: S1 Fig — (TIF) [file pntd.0012373.s003.tif]
